# Total Hip Arthroplasty in Teenagers: A Systematic Literature Review

**DOI:** 10.1097/BPO.0000000000002578

**Published:** 2023-11-29

**Authors:** Jens P. te Velde, George S. Buijs, Matthias U. Schafroth, Rachid Saouti, Gino M.M.J. Kerkhoffs, Arthur J. Kievit

**Affiliations:** * Department of Orthopedic Surgery and Sports Medicine, Amsterdam UMC, University of Amsterdam; † Amsterdam Movement Sciences, Musculoskeletal Health, Amsterdam, The Netherlands

**Keywords:** systematic review, total hip arthroplasty (THA), teenagers, children

## Abstract

**Background:**

Total hip arthroplasty (THA) in teenagers is generally avoided. Nevertheless, recent THA procedures in a very young patient show improved functional outcomes and implant survival, resulting in lower revision rates. This review aims to present an overview of the available literature on THA in teenagers and to provide evidence to inform caregivers.

**Methods:**

In this systematic review, studies required a primary THA method and a teenage patient population. Studies must report at least one of the following outcome measures: functional outcomes, implant survival, and complications. In addition, demographic and surgical data were collected.

**Results:**

Sixteen studies were analyzed, including 2040 patients and 2379 hips, with an average 7.7-year follow-up. The mean patient age was 18 years, with an average revision rate of 11.7%. The overall average relative improvement of the 2 most frequently used patient-reported (functional) outcome measures were 84.3 and 92.3% at the latest follow-up. Prosthesis, or liner loosening, was the cause of revision in 50.2% of the cases. Loosening was the most frequent complication (14.8%), together with prosthesis/liner wear (14.8%). Cementless fixation (70.7%), ceramic-on-ceramic articulation (34.7%), and the posterior surgical approach (82.3%) were the most applied techniques.

**Conclusions:**

The functional outcomes after THA in teenagers improved at follow-up. The average revision rate is relatively high, especially in the pre-1995 studies, with post-1995 studies reporting similar revision rates to the adult patient group. Research to further improve implant survival as well as the ease of revisions in teenagers is needed.

**Level of evidence:**

Level III—systematic review.

Total hip arthroplasty (THA) is an elective surgical procedure with excellent results regarding physical function, pain, (implant) survival, and mobility. THA is one of the most commonly performed procedures in orthopaedic surgery.^1^ Currently, THA is mainly performed on elderly patients, with an average age of around 65 years.^2^ Patients below 50 or 55 years old are considered young in this discipline. It is not clear how frequently THA’s are done by teenagers. As an indication of the scarcity, only 0.08% of all THA procedures in Australia were performed on patients under 21 (1999 to 2012).^3^ Reportedly, the results in the teenage group have yet to be proven equal to the results in older patients.^4^

THA is generally avoided by teenagers. Orthopaedic surgeons are historically hesitant to consider THA for skeletally immature patients due to many technical difficulties: distorted and complex hip anatomy, perceived high risk of failure, and the awareness of required revisions in the future due to limited longevity.^5^ Therefore, conservative or alternative (surgical) treatment is often considered to postpone a THA for adolescent patients.^6^ In some cases, this further deteriorates the hip joint through the deforming disposition that can characterize juvenile hip disease.^7^

Since THA in teenagers is so infrequently performed, relevant research regarding the outcome at follow-up is lacking in the current-day available literature. Moreover, underage subjects are often grouped with older subjects, which decreases the applicability of the outcomes of these studies for this very young patient group.^8^ A recent descriptive observational study reported the revision rate to be inversely proportional to the patient’s age.^9^ Nevertheless, in recent literature, THA in the very young patient appears to perform very well.^10^ It is believed that specific improvements over the last decades in implant fixation, articulation, or surgical approach play a role in these trends.^10^

It is hypothesized that THA outcomes in teenagers are close to or equal to those in the regular older patient group. Therefore, this review aims to present a complete overview of available literature on THA in teenagers to provide evidence to inform caregivers in their consideration as a tool for shared decision-making.

## METHODS

A systematic literature search in the PubMed (MEDLINE) database was conducted. The main concepts were (total hip arthroplasty OR total hip replacement) AND (young OR children OR teenagers OR juvenile) (Appendix S1, Supplemental Digital Content 1, http://links.lww.com/BPO/A674). All potential articles were collected, and duplicates were removed. Articles were independently screened on title and abstract for eligibility by 2 reviewers using Rayyan.^11^

The reference lists of included and excluded studies were screened for additional undiscovered studies in the initial literature search. Subsequently, full-text screening was performed by the 3 independent reviewers based on prespecified inclusion and exclusion criteria. All reviewer disagreements in the selection process were resolved by consensus-based discussion.

The studies required a primary THA method and must have used original data. The studies must consist of a teenage patient population, with a range of a minimum of 8 and a maximum of 24 years of age allowed, if the average age is between 11 and 19. Case reports were excluded. Articles that primarily examine patients with malignant bone diseases were excluded since the limited survivorship of the patients and the anticipation of required revisions with oncologic indications could make these patients less comparable.^12,13^ A restriction was placed on articles published before 2000 due to the expected improvements in THA procedures in the last century. Articles must be written in Dutch or English. Articles with a loss of follow-up >20% were excluded, as this is common in orthopaedic research, despite the arbitrariness.^14^

Primarily, the following outcome measures were collected and evaluated: survival of the prostheses (revision rate), complications (eg, dislocations), and functional outcomes [eg, Harris hip score (HHS)]. In addition, demographic and surgical data (mean patient age, indication for primary THA, fixation, articulation, and surgical approach) were collected and described. When fractions are presented in percentages, missing data is included in the denominator, unless stated otherwise.

Two independent reviewers (J.V. and G.B.) used the Methodological Index for Nonrandomized Research instrument to rate the methodological quality of the research.^15^ Maximum scores are 16 for noncomparative, nonrandomized studies and 24 for comparative, nonrandomized studies. Items were given a score of “0” for not being reported, “1” for inadequate reporting, and “2” for good reporting. All differences were resolved by dialog between the 2 separate reviewers after an independent examination. If there was still a difference of opinion, the conclusion reached by a third investigator (A.K.) was final.

### Revision rate and complications

This study distinguished complications requiring revision (“causes of revision”) and complications not requiring revision since both are unforeseen and undesirable events, while they have different implications. The revision or implant survival rates and the concomitant time and cause of the revision were collected, with only the first revision per hip accumulated. Hips reportedly requiring or awaiting revision could not be enclosed, but their cause for future revision could be included as complications. The exact frequencies of complications must be specified in the studies to be counted. No distinction was made in acetabular or femoral loosening or wear; if either component of both is loose, the cause of revision or complication was reported as “loosening.” Some of the included studies refrained from reporting findings on radiographs, such as radiolucencies. Next to symptomatic radiolucencies, we included radiolucent lines wider than 2 mm as “loosening” in studies that refrained from reporting radiographical loosening.^16^

### Functional Outcomes

Most studies evaluating functional outcomes after hip procedures used one of the following patient-reported outcome measures (PROMs); the HHS, Hip Disability and Osteoarthritis Outcome Score for Joint Replacement, and Merle d’Aubigné and Postel Score (Merle d’Aubigné). As it is likely that different PROM questionnaires were used in the included studies and scores may not be directly comparable, it was decided to express PROMs as a percentage of the maximum scale score and as percentages of score improvement.

### Surgical Data

Several fixation possibilities were acknowledged in the data: uncemented (uncemented acetabular cup, uncemented femoral stem), cemented (cemented cup, cemented stem), hybrid (uncemented cup, cemented stem), and reverse hybrid (cemented cup, uncemented stem). When studies did not specify which cup and stem were combined, the minimal fixation combinations were calculated, while the remainder was reported as unknown.

Articulation materials were collected in the data as well. Ceramic femoral heads usually articulate with: (1) ceramic (ceramic-on-ceramic, CoC), (2) metal (ceramic-on-metal, CoM), or (3) polyethylene acetabular cups liners (ceramic-on-polyethylene, CoP). Metal femoral heads were generally placed with: (1) metal (metal-on-metal, MoM) or (2) polyethylene (metal-on-polyethylene, MoP) acetabular cup liners. The specific types of polyethylene, namely ultra-high-molecular weight polyethylene or the more recently implemented highly cross-linked polyethylene (XLPE), were distinguished.

Adjacent to the fixation and articulation, the type and frequency of the applied surgical approach were collected for the included articles.

## RESULTS

**FIGURE 1:**
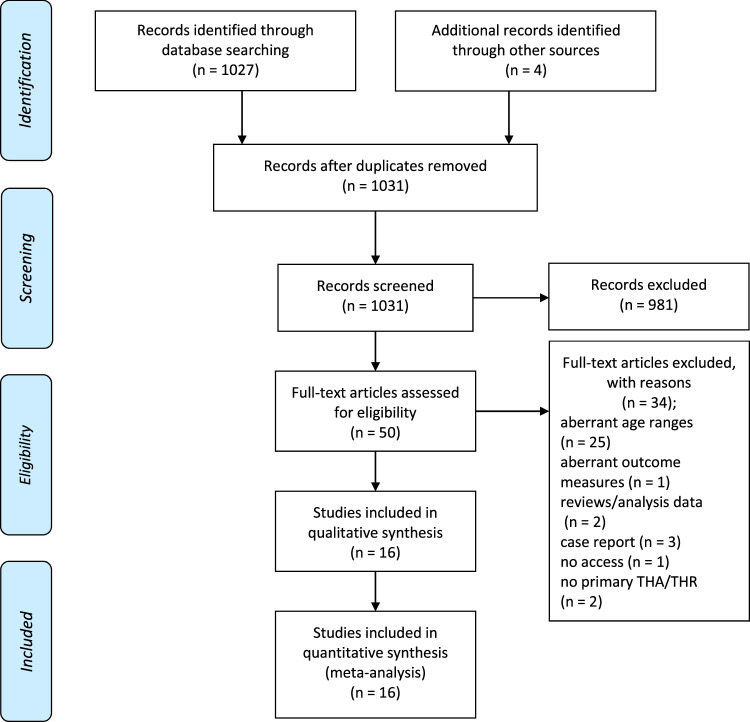
Flow diagram of the systematic search. THA indicates total hip arthroplasty, THR, total hip replacement.

Sixteen studies were identified, including 2060 patients and 2409 hips.^5,7–10,17–27^ The indication of primary THA of 2388 hips could be collected because several studies presented only the indication per patient instead of per hip, even for bilateral patients (Table 1). Pediatric hip disorders were the most frequent indication for primary THA (n=1119, 46.9%). The mean age of the included patients was 18 years (8 to 24 y) at the time of surgery. The total patient population after the loss of follow-up after primary THA consisted of 2040 patients and 2379 hips. The follow-up period, reported by all but one study, varied through the included articles, with a mean of 7.7 years (0.7 to 38 y). A flow diagram summarizing the data collection and tables presenting the included articles were enclosed (Fig. 1, Tables 1–4).

**TABLE 1:** Indications for Primary Total Hip Arthroplasty

Indications (n=2388)	n (%)
Pediatric hip disorder	1119 (46.9)
AVN	465 (19.5)
DDH	219 (9.2)
LCPD	69 (2.9)
SUFE	76 (3.2)
n/s	290 (12.1)
Arthritis	524 (21.9)
OA	214 (9.0)
Post-	136 (5.7)
JIA	130 (5.4)
JRA	32 (1.3)
Septic	12 (0.5)
Other	366 (15.3)
Systemic inflammatory disease (n/s)	234 (9.8)
Trauma/bone fracture	75 (3.1)
Infection	23 (1.0)
Earlier surgery	13 (0.5)
Ankylosing spondylitis	11 (0.5)
Protrusio acetabuli	10 (0.4)
Other unfrequent causes (n<10)	379 (15.9)

AVN indicates avascular necrosis of the femoral head; DDH, developmental dysplasia of the hip; JIA, juvenile idiopathic arthritis; JRA, juvenile rheumatoid arthritis; LCPD, Legg-Calve-Perthes disease; n/s, not specified; OA, osteoarthritis; Post-, post-trauma/postinfection arthritis; Septic, septic arthritis; SUFE, slipped upper femoral epiphysis.

**TABLE 2:** Causes of Revision and Complications for Primary Total Hip Arthroplasty.

	Blood related	Bone fracture	Contracture	Dislocation	Heterotopic ossification	Instability/subluxation	Loosening	Nerve injury	Noise generation	Pain (only)	Perioperative problem	Prosthesis fracture	Prosthesis/liner wear	Severe osteolysis	Wound problem	Not listed/unknown	Total
Causes of revision	0	6	0	14	0	1	140	0	1	7	1	2	30	13	18	46	279
%	–	2.2	–	5.0	–	0.4	50.2	–	0.4	2.5	0.4	0.7	10.8	4.7	6.5	16.5	100
Complications	8	2	1	7	1	5	13	9	5	0	6	0	13	10	8	0	88
%	9.1	2.3	1.1	8.0	1.1	5.7	14.8	10.2	5.7	0	6.8	0	14.8	11.4	9.1	0	100

Blood-related indicates perioperative blood transfusion/hematoma/pulmonary embolism; bone fracture, periprosthetic fracture; nerve injury, femoral/peroneal/sciatic injury or palsy/foot drop/(abductor) lurch/Trendelenburg; wound problem, wound dehiscence/delayed wound healing/(postoperative) infection.

**TABLE 3:** Functional Outcomes and Revision Data at Follow-up

				Age	Follow-up (y)	PROMs	Preop		Postop		Improvement	Revisions
References	Time period	Patients	Hips	Mean (min-max)	Mean/median (min-max)	Type	% of max	Mean (min-max)	% of max	Mean (min-max)	Abs. (%)	No. (%)
Bessette et al.^17^	1975-1990	11	15	16.5 (10-20)	13.6 (10-25)	HHS			64.5	64.5 (34.2-97.2)		4 (26.7)
Buddhdev et al.^18^	2003-2017	51	60	16.7 (12-19)	9.3 (2.3-16.8)	OHS			91.7	44 (31-48)		2 (0.03)
Daurka et al.^19^	1995-2005	33	49	14.4 (10-16)	10.5 (6-15)	HSS	32	12.8 (6-22)	80	32 (22-40)	19.2 (150)	13 (26.5)
Finkbone et al.^20^	1998-2008	19	24	16.4 (12-20)	4.3 (2.1-10.3)	mHHS	47.7	47.7 (37-59)	93.4	93.4 (66-100)	45.7 (95.8)	1 (0.04)
Halvorsen et al.^10^	1995-2016	747	881	18 (9-21)	n/s (n/s-20)	n/s						118 (13.4)
Hannouche et al.^21^	1979-2013	71	88	17.3 (13.2-20)	8.8 (2-34.4)	Merle d’Aubigné	56.1	10.1	97.8	17.6	7.5 (74.3)	17 (19.3)
Kahlenberg et al.^22^	1982-2018	113	129	17 (8-20)	13.2 (2-38)	HOOS, JR.			84.6	84.6		27 (20.9)
Kamath et al.^5^	n/s	18	21	18 (13-20)	4.1 (2.1-7.4)	HHS	43.6	43.6 (11-83)	83.6	83.6 (63-100)	40 (91.7)	1 (4.8)
Kitsoulis et al.^23^	1984-2002	10	20	15.8 (13-24)	9.2 (2-20)	mMerle d’Aubigné	48.9	8.8	100	18(18)	9.2 (104.5)	2 (10)
Luceri et al.^24^	2009-2018	10	12	17 (14-20)	3.3 (0.7-10.1)	HHS	36.1	36.1 (11.55-59)	94	94 (90-96)	57.9 (160.4)	1 (8.3)
Metcalfe et al.^9^	2003-2017	703	769	18 (13-20)	5.1 (n/s)	n/s						35 (4.6)
Pallante et al.^7^	1998-2016	78	91	17 (11-20)	8 (2-18)	mHHS			92	92 (54-100)		2 (2.2)
Restrepo et al.^25^	1993-2003	21	29	17.6 (13.5-20)	6.6 (4.2-10)	HHS	51.9	51.9 (40.1-82.3)	77.3	77.3 (60-99)	25.4 (49.9)	1 (3.4)
Tsukanaka et al.^26^	1987-2010	111	132	17 (11-19)	14 (3-26)	HHS			83	83 (15-100)		39 (29.5)
Van de Velde et al.^8^	2005-2016	18	24	14.6 (11-16)	3.8 (1-8)	mMerle d’Aubigné	34.4	6.2	98.3	17.7	11.5 (185.5)	0 (0)
Wroblewski et al.^27^	1969-2001	26	35	17.9 (12-19)	15.9 (2.3-34)	mMerle d’Aubigné			79.8	14.4		16 (45.7)

(m)HHS indicates (modified) Harris Hip score; (m)Merle d’Aubigné, (modified) Merle d’Aubigné and Postel score; HOOS, JR, hip disability and osteoarthritis outcome score for joint replacement; HSS, hospital for special surgery score; OHS, Oxford hip score; PROMs, patient-reported outcome measures.

**TABLE 4:** Demographic and Surgical Data for Primary Total Hip Arthroplasty.

					Cementation (# hips)					Articulation (# hips)							
References	Time period	Patients	Hips	Baseline	Uncemented	Hybrid	Cemented	Reverse hybrid	n/s	CoC	CoM	CoP	MoM	MoP	n/s	Polyethylene type	Surgical approach (# hips)
Bessette et al.^17^	1975-1990	11	15		11	2	2								15		n/s
Buddhdev et al.^18^	2003-2017	51	60		60	0	0			60							Posterior (60)
Daurka et al.^19^	1995-2005	33	49	35 pts 52 hips	52	0	0			23				29		n/s	Anterolateral (16) Posterior (36)
Finkbone et al.^20^	1998-2008	19	24							24							Anterolateral (2) Posterior (22)
Halvorsen et al.^10^	1995-2016	747	881		659	36	62	78	46	97	1	189	145	284	165	CoXLPE (135) MoXLPE (206)	Posterior (418) Not posterior (262) Unknown (201)
Hannouche et al.^21^	1979-2013	71	88	83 pts 105 hips	72	29			4	105							Lateral (7) Posterior (98)
Kahlenberg et al.^22^	1982-2018	113	129		113	14	2			10	2	58	2	57		n/s	n/s
Kamath et al.^5^	n/s	18	21		20	1	0			14			1	6		MoXLPE (6)	Posterolateral (21)
Kitsoulis et al.^23^	1984-2002	10	20		10	10	0								20		Lateral (2) Posterolateral (18)
Luceri et al.^24^	2009-2018	10	12		12	0	0			7		5				n/s	Anterior (3) Lateral (4) Posterolateral (5)
Metcalfe et al.^9^	2003-2017	703	769		451		73		245	438		116	30	68	117	n/s	n/s
Pallante et al.^7^	1998-2016	78	91		83	8	0			53		10		28		CoXLPE (10) MoXLPE (28)	Anterolateral (16) Posterior (75)
Restrepo et al.^25^	1993-2003	21	29	25 pts 35 hips	35	0	0			2		22		11		CoXLPE (22)	Anterolateral (35)
Tsukanaka et al.^26^	1987-2010	111	132		111			8	13	4		72	3	45	8	UHMWPE (84) XLPE (33)	n/s
Van de Velde et al.^8^	2005-2016	18	24		24	0	0							24		n/s	Lateral (24)
Wroblewski et al.^27^	1969-2001	26	35	28 pts 39 hips	0	0	39					39				UHMWPE (39)	n/s
		2040	2379	2060 pts 2409 hips	1713	100	178	86	308	837	3	511	181	552	325	CoXLPE (167) MoXLPE (240) UHMWPE (123) XLPE (33)	Anterior (3) Anterolateral (69) Lateral (37) Posterior (709) Posterolateral (44) Unknown n/s (1547)

CoC indicates ceramic-on-ceramic; CoM, ceramic-on-metal; CoP, ceramic-on-(highly cross-linked) polyethylene; CoXLPE, ceramic-on-highly cross-linked polyethylene; hybrid, acetabular cup uncemented, femoral head cemented; reverse hybrid, acetabular cup cemented, femoral head uncemented; MoM, metal-on-metal; MoP, metal-on-(highly cross-linked) polyethylene; MoXLPE, metal-on-highly cross-linked polyethylene; UHMWPE, ultra-high-molecular weight polyethylene; XLPE, highly cross-linked polyethylene.

The first 2 authors reached a consensus on each case’s assessment of the studies’ methodological quality. All included studies had Methodological Index for Nonrandomized Research Ratings ranging from 10 to 12 (Appendix S2, Supplemental Digital Content 2, http://links.lww.com/BPO/A675).

### Revision Rate and Complications

**FIGURE 2:**
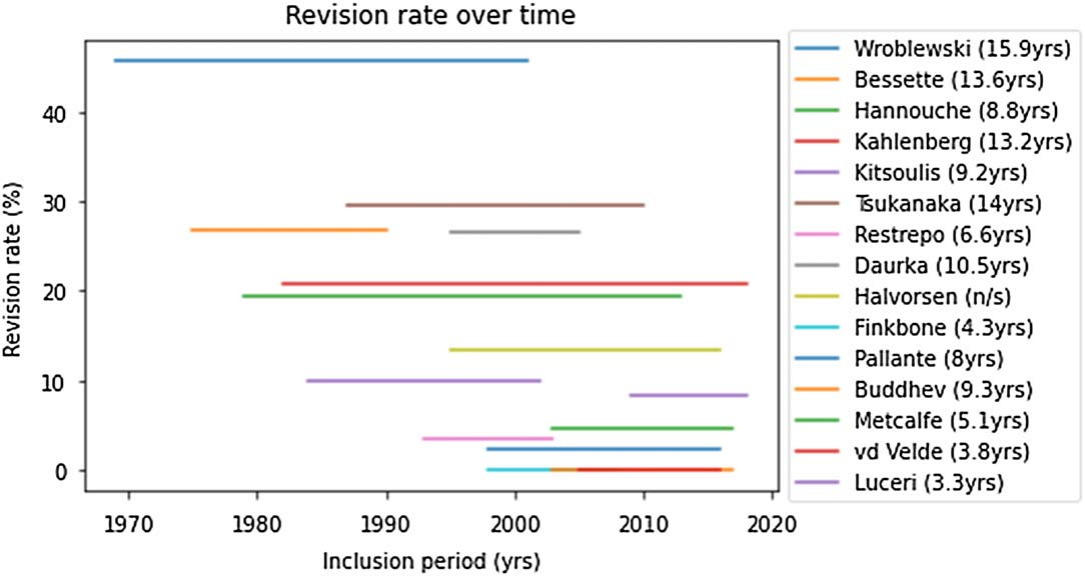
Revision rate in relation to the study inclusion periods. The included studies are shown by their first author with the average follow-up in years. The article by Kamath et al.^5^ refrained from reporting inclusion periods and is not included in this figure.

All 16 studies reported the revision or prosthesis survival rate in their results, with 279 revision procedures in all (Table 2). Accordingly, a total revision rate of 11.7% (279/2379) could be established in the mean follow-up period of 7.7 years, with rates varying from 0 to 45.7% between included studies. Figure 2 shows the revision rate in relation to the inclusion periods, regardless of the average follow-up of the studies as listed in Table 3. Causes of revision surgery were available in 233 of 279 revisions (Table 2). The leading cause was loosening (50.2%), followed by wear (10.8%). In total, 88 complications were collected from the included studies (Table 2). Loosening (14.8%) and wear (14.8%) were reported with the highest frequency, corresponding with the causes of revision. Five studies refrained from reporting complications or reported no occurrence.^9,10,18,22,25^

### Functional Outcomes

Between all studies, various PROMs were reported (Table 3). HHS scores were reported in five studies, and the modified HHS was applied in 2 studies. In the mHHS studies, a multiplier was used to retain the original maximum score of 100.^28^ For this reason, the 4 studies that reported HHS or mHHS scores both preoperatively and at the latest follow-up were compared and showed an average improvement preoperatively as opposed to postoperatively of 39.2 points (+84.3%).^5,20,24,25^ Of the 7 studies that applied the (m)HHS, the mean score at the latest follow-up was poor in one (64.5), fair in 1 (77.3), good in 2 (83, 83.6), and excellent in 3 (92, 93.4, and 94), according to the definitions of Harris.^5,7,17,20,24–26,29^ The weighted average of the total (m)HHS group was 46.5% preoperatively as opposed to 85.4 at the latest follow-up and 85.7 when only the 4 studies containing preoperative results were included. Since the maximum (m)HHS score is 100, the corresponding percentages of the maximum score were identical.

The Merle d’Aubigné score was reported in 2 closely correlated designs, namely the original score in 1 study and the modified Merle d’Aubigné and Postel score (mMerle d’Aubigné) in 3 studies.^8,21,23,27,30^ Therefore, the outcomes from preoperatively and the latest follow-up of the 3 studies that reported both were compared collectively and showed an average improvement of 8.5 points (+92.3%).^8,21,23^ The weighted average of the total (m)Merle d’Aubigné group was 9.2 preoperatively as opposed to 17.0 at the latest follow-up and 17.7 when only the 3 studies with preoperative results were included. Since the maximum score of the (m)Merle d’Aubigné score is 18, the corresponding percentages of the maximum score were 51.1% preoperatively and 94.4% and 98.3% at the latest follow-up. The remainder of the studies either refrained from reporting preoperative PROMs or used incomparable PROMs.

### Surgical Data

Surgical data were reported in Table 4. Most implants were placed by cementless fixation (71.8%). Due to the differentiation in acetabular and femoral fixation in some studies and the unknown fixation in others, it was not possible to present exact quantifications. The fixation techniques used were not reported in 342 hips (14.2%). The most frequent bearing was CoC (34.7%). Of 325 hips (13.5%), the articulation was not reported. Of the 1063 hips that used polyethylene, ultra-high-molecular weight polyethylene was reported in 123 hips and XLPE in 440 hips. The surgical approach was reported in only 862 of 2409 hips (35.8%), with the majority being the posterior approach (29.4%).

## DISCUSSION

This study aimed to present a complete overview of the available literature on THA in teenagers. Sixteen studies with a total of 2379 hips in 2040 patients were reviewed.

### Revision Rate and Complications

The reported revision rate in this study (11.7%) was relatively high compared with the benchmark 10-year revision rate in older patients (median age 70.4 y) of under 5%.^31^ However, due to the more active lifestyle of younger teenage patients, higher prosthesis demands could be expected, with correspondingly increased revision rates.^32^ It was also suggested that more recent studies show improved revision rates because they refrained from using earlier and inferior bearing surface material.^10^ Due to the long inclusion periods, it was challenging to differentiate between older and more recently commenced studies.

Nevertheless, a remarkable difference in revision rates could be displayed when studies were split in their starting time. Studies that commenced before the cutoff point of 1995 had an average revision rate of 23.7% in 12.2 years, compared to 5.2% in 5.8 years for studies that started in 1995 or later. When the cutoff point is set at 2000, the difference was 19.9% in 11.1 years for 20th century studies and no less than 4.4% in 5.3 years for studies that started more recently. Since the follow-up periods differed, comparing studies before or after the 2 cutoff points might be incorrect. However, the revision rate for reviews commenced after 2000 (4.4%) was compliant with the earlier-mentioned benchmark 10-year revision rate of 5% in the older patients.^31^ This suggested that the recent teenage THA procedures are coming closer to the older population regarding short-term revision rates. The revision rate in relation to the inclusion periods is presented in Figure 2. This review is susceptible to an underestimation of the revision rate after the loss of follow-up, especially with the small cohorts in some of the included studies. This should be mentioned regardless of the exclusion of studies with a loss of follow-up >20%.

Outcomes and complications as seen on radiographs were challenging to interpret due to varying definitions of outcomes and a lack of radiographic analyses in multiple cases, and were therefore not separately enclosed in this review, despite their clinical relevance.

Both as causes of revisions and complications, this review reported a 0.88% dislocation rate, compared with 3.24% in older patients.^33,34^ More research must be conducted to establish whether this reported difference is retained over a long-term follow-up period.

### Functional Outcomes

In a recent study in which 109 patients (mean age of 62.1 y) underwent a THA following an enhanced recovery program, the absolute improvement score was 39.0 (HHS), similar to the average absolute improvement of 39.2 reported in this review [(m)HHS].^35^

The included studies that applied (m)Merle d’Aubigné showed a relatively better percentage of the maximum scores at the latest follow-up compared with the included (m)HHS studies (94.4% compared with 85.4%). The (m)Merle d’Aubigné preoperative scores were higher than the (m)HHS preoperative scores as well, but this difference was smaller (51.1% to 46.5%). Explanations for these distinctions were unknown but may be explained by the unprecedented collective comparison of the 2 HHS scores and the 2 Merle d’Aubigné scores in this study. Even though the scores and their corresponding modified versions are reported to have excellent correlations, it was unclear whether the reported improvement scores were obtained correctly.^30^

Of the applied PROMs, (m)HHS and (m)Merle d’Aubigné were reported with the highest frequency and are widely used in orthopaedic research for examining hip function.

In both PROMs, the ceiling effect had a repeated occurrence, which could harm their validity and usefulness, especially when the percentages of the maximum score were used.^30,36^

### Surgical Data

The results show that most hips were fixated through the uncemented method, which complies with the current trend. A possible explanation could be that for the fixation choice in very young patients with a high chance of revisions in their lifetime, future revisions without challenges in the removal of cement might be preferable.^4^

The high frequency of CoC articulation used in this study was surprising, considering the declining trend of CoC in the last decade throughout all age groups.^4,37^ Ceramic bearings might be applied regularly to very young patients due to the reported exceptional wear rates.^38^ XLPE use has resulted in a considerable wear rate and osteolysis decrease and improved implant survival in 10 years of follow-up. Distinctions in polyethylene type have great significance and must be covered in further research, as they were difficult to describe in this study due to ambiguity in reporting.^39,40^

The posterior approach was applied to most hips in this review, but this data is biased due to a lack of information. By virtue of this review, there are no signs that a specific surgical approach is advantageous over the others in this young patient population.

### Future Research

The PROMs and revision rate are all established for an average follow-up time of only 7.7 years, with the most extended follow-up of 15.9 years.^27^ Therefore, the outcomes in around 20 to 30 years of long-term follow-up are unclear and need to be examined in further research. The functional results of long-term follow-up are highly relevant for this young patient population. Future studies should also conduct correlation analyses for surgical data with regards to the survival rate, complications, and functional outcomes.

Only 6 of the included studies reported patient satisfaction after THA, even though this is increasingly emphasized in orthopaedic literature to reduce the proportions of virtually exclusively objective measurement outcomes.^7,17,20–23,41^ The anticipated improvements in the quality of life should be reported in future research. Similar to this is work participation, a specifically interesting outcome for these patients who have yet to begin their careers or continue their education. However, this outcome is presented in only 3 of the included reviews.^8,21,25^ Modern adult’s study shows promising work participation rates of around 86%, which should be established for the teenage patient population as well.^42^

## CONCLUSIONS

The average revision rate is relatively high, especially in the pre-1995 studies, with post-1995 studies reporting revision rates coming closer to the adult patient group. The functional outcomes after THA in teenagers improved preoperatively compared with follow-up, regardless of follow-up duration. Yet, these results should be interpreted with caution due to the long inclusion periods causing variability and the lack of long-term follow-up. Therefore, further research is needed to assist clinicians in shared decision-making for this specific patient group.

## Supplementary Material

SUPPLEMENTARY MATERIAL
