# Supplementary material for: Total Hip Arthroplasty in Teenagers: A Systematic Literature Review
Source: J Pediatr Orthop. 2023 Nov 29;44(2):e115–23. doi: 10.1097/BPO.0000000000002578 (PMC10766098; doi:10.1097/BPO.0000000000002578)
Supplement: SUPPLEMENTARY MATERIAL [file bpo-44-e115-s001.docx]

# Appendix

Appendix S1

| (total hip arthroplasty OR total hip replacement) AND (young OR children OR teenagers OR juvenile) |
| --- |
| Filters applied:^1^  Text availability: Free full text, Full text  Language: Dutch, English  Age: Child: birth-18 years, Adolescent: 13-18 years, Young Adult: 19-24 years |

*Appendix S1: PubMed search strategy*

*^1^: filtered for publication year (2000 - )*
