# Supplementary material for: Total Hip Arthroplasty in Teenagers: A Systematic Literature Review
Source: J Pediatr Orthop. 2023 Nov 29;44(2):e115–23. doi: 10.1097/BPO.0000000000002578 (PMC10766098; doi:10.1097/BPO.0000000000002578)
Supplement: SUPPLEMENTARY MATERIAL [file bpo-44-e115-s002.docx]

Appendix S2

| ***Quality assessment non-randomized trials*** | Bessette^17^ | Buddhdev^18^ | Daurka^19^ | Finkbone^20^ | Halvorsen^10^ | Hannouche^21^ | Kahlenberg^22^ | Kamath^5^ | Kitsoulis^23^ | Luceri^24^ | Metcalfe^9^ | Pallante^7^ | Restrepo^25^ | Tsukanaka^26^ | Vd Velde^8^ | Wroblewski^27^ |
| --- | --- | --- | --- | --- | --- | --- | --- | --- | --- | --- | --- | --- | --- | --- | --- | --- |
| *A clearly stated aim* | 2 | 2 | 2 | 2 | 2 | 2 | 2 | 2 | 1 | 1 | 2 | 2 | 2 | 2 | 2 | 2 |
| *Inclusion of consecutive patients* | 2 | 2 | 2 | 2 | 2 | 2 | 2 | 2 | 2 | 2 | 2 | 2 | 2 | 2 | 2 | 2 |
| *Prospective data collection* | 0 | 2 | 2 | 2 | 2 | 2 | 2 | 2 | 2 | 2 | 2 | 2 | 2 | 2 | 2 | 2 |
| *Endpoints appropriate to the aim of the study* | 2 | 2 | 2 | 2 | 2 | 2 | 2 | 2 | 2 | 2 | 2 | 2 | 2 | 2 | 2 | 2 |
| *Unbiased assessment of the study endpoint* | 0 | 0 | 0 | 0 | 0 | 0 | 0 | 0 | 0 | 0 | 0 | 0 | 0 | 0 | 0 | 0 |
| *A follow-up period appropriate to the aims of study* | 2 | 2 | 2 | 2 | 2 | 2 | 2 | 2 | 2 | 1 | 1 | 1 | 2 | 2 | 2 | 2 |
| *Less than 5% loss to follow-up* | 2 | 2 | 1 | 1 | 1 | 1 | 1 | 2 | 2 | 2 | 2 | 1 | 1 | 1 | 2 | 1 |
| *Prospective calculation of the sample size* | 0 | 0 | 0 | 0 | 0 | 0 | 0 | 0 | 0 | 0 | 0 | 0 | 0 | 0 | 0 | 0 |
| *An adequate control group* |  |  |  |  |  |  |  |  |  |  |  |  |  |  |  |  |
| *Contemporary groups* |  |  |  |  |  |  |  |  |  |  |  |  |  |  |  |  |
| *Baseline equivalence of groups* |  |  |  |  |  |  |  |  |  |  |  |  |  |  |  |  |
| *Adequate statistical analyses* |  |  |  |  |  |  |  |  |  |  |  |  |  |  |  |  |
| **Total score** | 10 | 12 | 10 | 11 | 11 | 11 | 11 | 12 | 11 | 10 | 11 | 10 | 11 | 11 | 12 | 11 |

*Appendix S2: Assessment of Methodological Quality using the MINORS tool*
